# Invasive lobular and ductal breast carcinoma differ in immune response, protein translation efficiency and metabolism

**DOI:** 10.1038/s41598-018-25357-0

**Published:** 2018-05-08

**Authors:** Tian Du, Li Zhu, Kevin M. Levine, Nilgun Tasdemir, Adrian V. Lee, Dario A. A. Vignali, Bennett Van Houten, George C. Tseng, Steffi Oesterreich

**Affiliations:** 1 Womens Cancer Research Center, UPMC Hillman Cancer Center, Magee Womens Research Institute, Pittsburgh, PA 15213 USA; 2 School of Medicine, Tsinghua University, Beijing, 100084 China; 3 Department of Biostatistics, University of Pittsburgh, Pittsburgh, PA 15213 USA; 4 Department of Pathology, University of Pittsburgh, Pittsburgh, PA 15213 USA; 5 Department of Pharmacology & Chemical Biology, University of Pittsburgh, Pittsburgh, PA 15213 USA; 6 Department of Immunology, University of Pittsburgh School of Medicine, Pittsburgh, PA 15213. Tumor Microenvironment Center, UPMC Hillman Cancer Center, Pittsburgh, PA 15232 USA; 7 Department of Computational & Systems Biology, Pittsburgh, PA 15213 USA

## Abstract

Invasive lobular carcinoma (ILC) is the second most common histological subtype of breast cancer following invasive ductal carcinoma (IDC). ILC differs from IDC in a number of histological and clinical features, such as single strand growth, difficulty in detection, and frequent late recurrences. To understand the molecular pathways involved in the clinical characteristics of ILC, we compared the gene expression profiles of luminal A ILC and luminal A IDC using data from TCGA and utilized samples from METABRIC as a validation data set. Top pathways that were significantly enriched in ILC were related to immune response. ILC exhibited a higher activity of almost all types of immune cells based on cell type-specific signatures compared to IDC. Conversely, pathways that were less enriched in ILC were related to protein translation and metabolism, which we functionally validated in cell lines. The higher immune activity uncovered in our study highlights the currently unexplored potential of a response to immunotherapy in a subset of patients with ILC. Furthermore, the lower rates of protein translation and metabolism - known features of tumor dormancy - may play a role in the late recurrences of ILC and lower detection rate in mammography and PET scanning.

## Introduction

Invasive lobular carcinoma (ILC) and invasive ductal carcinoma (IDC) are the two main histological subtypes of breast cancer. ILC accounts for 10–15% of all breast cancers^1,2^ and is characterized by small, round tumor cells growing in stroma in a discohesive single-file pattern^3^. In comparison with IDC, ILC is more difficult to detect by standard imaging techniques like mammography and ^18^F-FDG-PET^3–8^. In general, ILC is detected in patients at an older age and at a more advanced stage than IDC^9^. Compared to stage/grade-matched IDC, patients with ILC display relative late recurrences and worse long-term survival^10–13^. We and others have described a unique metastatic dissemination of ILC, including decreased metastases to visceral organs, and increased metastases to ovary, and the gastrointestinal tract^3,14–16^. While endocrine therapy and chemotherapy are frequently used to treat both ILC and IDC, patients with ILC may have lower response rates to neoadjuvant chemotherapy and slightly worse outcomes to tamoxifen compared to patients with IDC^17–19^. Although other novel therapeutic approaches such as immunotherapy are proving to be promising in a subset of breast cancers, especially in the triple negative subtype^20^, less data have been reported on the immune response in ILC, likely due to its generally understudied nature as a unique breast cancer subtype.

The main differences between the two histological subtypes is the lack of E-cadherin (CDH1) protein expression in ~90% of ILC^1,2,21^. ILC more often expresses estrogen receptor (ER) than IDC, with ~90% of ILC being ER positive. ILC also has high rates (50–70%) of progesterone receptor (PR)-positivity, but less than 10% express epidermal growth factor receptor 2 (HER2/ERBB2)^1,2,13,17,21,22^. While ILC generally exhibits lower Ki67 positivity than IDC^13,17,21^, it has a higher frequency of HER2 and HER3 mutations, PIK3CA mutations, FOXA1 mutations, ESR1 amplifications, and PTEN loss^1,2^. While there has been recent characterization of the differences between ILC and IDC at the genomic level^1,2,23^, differences in gene expression have not been sufficiently studied. Previous studies analyzing the transcriptomic profiles of ILC and IDC have been limited by small sample size^24–26^. Although recent large scale analyses by The Cancer Genome Atlas (TCGA)^2^ and Rational Therapy for Breast Cancer (RATHER)^27^ groups have identified different molecular subtypes within ILC based on mRNA expression data, gene expression differences between ILC and IDC remain largely unexplored^2,27^. Using *in silico* analyses and follow-up cell culture experiments, we show that ILC is characterized by unique immune signatures, decreased protein translation rates, and lower overall metabolism. Importantly, our results may help to explain some of the unique clinical features of ILC, and to guide further studies aimed at personalizing the diagnosis and treatment of this understudied histological subtype of breast cancer.

## Results

### Immune signatures are enriched in LumA ILC

To identify differentially expressed (DE) genes between IDC and ILC, we extracted publicly available RNA-Sequencing (RNA-Seq) data from The Cancer Genome Atlas (TCGA) (IDC: n = 774; ILC: n = 197) database^28^ and microarray data from the Molecular Taxonomy of Breast Cancer International Consortium (METABRIC) (IDC: n = 1548; ILC: n = 147) dataset^29^. We first assigned each sample to one of five intrinsic subtypes by PAM50^29^ (Supplementary Tables 1 and 2). The distributions of luminal A (LumA), luminal B (LumB), Normal-like, basal-like (basal) and HER2-enriched (HER2) molecular subtypes among the ILC samples were 81%, 9%, 7%, 1%, and 3% for TCGA tumors, and 40%, 20%, 26%, 3% and 6% for METABRIC tumors, respectively. While we don’t fully understand the reason for the difference in distributions of molecular subtypes comparing TCGA and METBARIC, it is likely a result of differences in the patient cohorts. For example, there are significant differences in stage distribution of the tumors, and age of the patients. In addition, there are also significant differences in cellularities of the tumors, which could have also affected PAM50 classifications. Given the small numbers of ILC samples in the Basal and HER2 groups, we limited our following expression analysis to the LumA, LumB, and Normal-like subtypes.

We performed DE gene analysis in the TCGA tumors using the DESeq2 algorithm^30^, which identified 11,611 and 7,033 genes based on Benjamini-Hochberge adjusted p-value (FDR) cut-offs of 0.05 and 0.001, respectively, for LumA tumors (Table 1). Fewer DE genes were identified in LumB and Normal-like tumors. Similar analysis of the METABRIC data failed to identify any DE genes in the LumB subtype, and relatively few in Normal-like tumors (Table 1), likely due to the small number of samples, and the lower dynamic range of microarray data as compared to RNA-Seq^31^. We therefore restricted our subsequent analyses to LumA tumors.

**Table 1:** Number of differentially expressed genes between ILC and IDC.

Number of differentially expressed (DE) Genes: ILC vs IDC					
TCGA			METABRIC		
PAM50	FDR < 0.05	FDR < 0.001	PAM50	FDR < 0.05	FDR < 0.001
**LumA** ILC: N = 159 IDC: N = 311	**11611**	**7033**	**LumA** ILC: N = 65 IDC: N = 533	**2469**	**0**
**LumB** ILC: N = 18 IDC: N = 202	**1415**	**110**	**LumB** ILC: N = 29 IDC: N = 401	**0**	**0**
**Normal** ILC: N = 13 IDC: N = 18	**1604**	**53**	**Normal** ILC: N = 38 IDC: N = 120	**621**	**0**

Number of differentially expressed genes with different cutoffs of p-value (0.05 and 0.001) in TCGA and METABRIC. Tumors within different breast cancer intrinsic molecular subtypes (PAM50) were analyzed separately.

**Figure 1:**
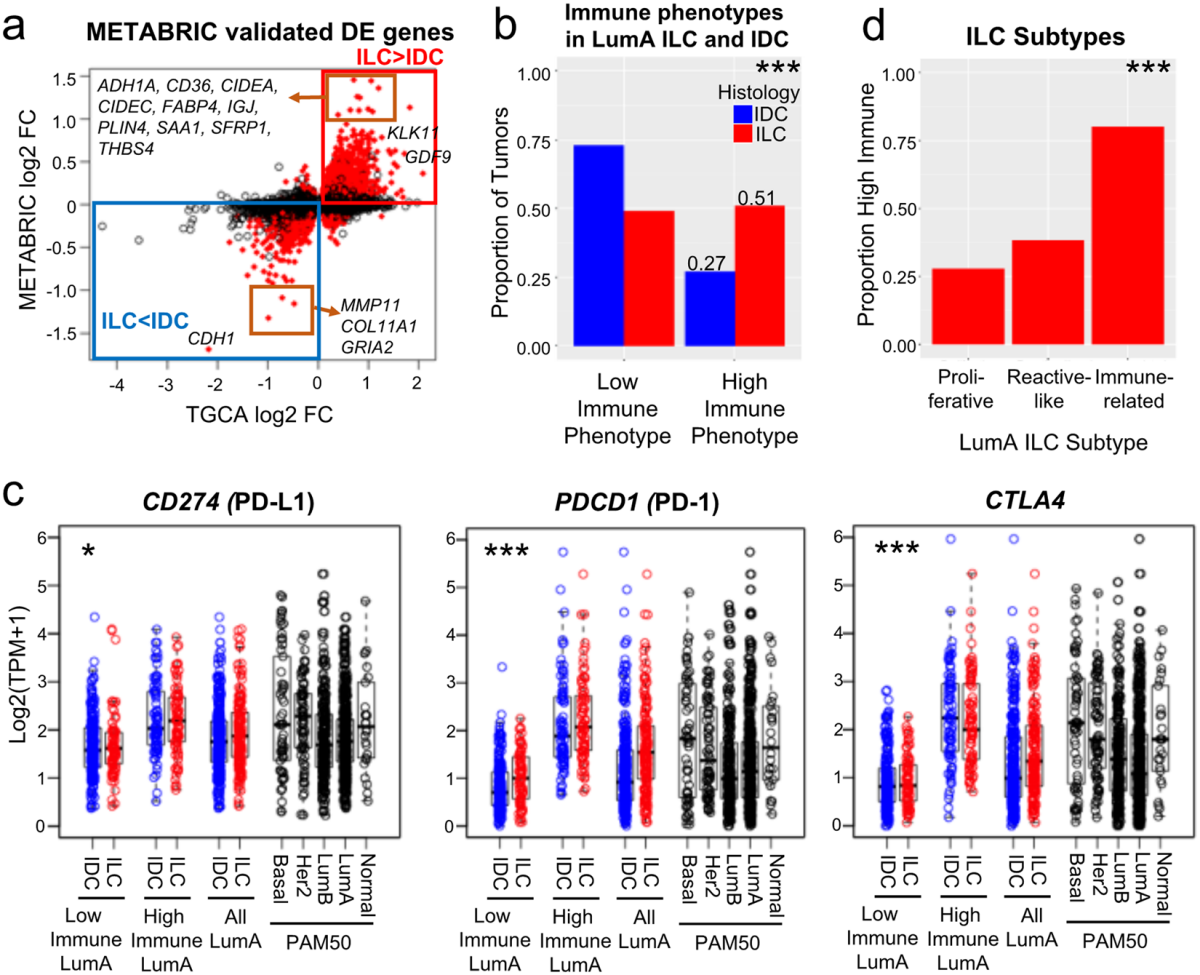
LumA ILC is enriched for immune cell infiltration and high immune-checkpoint gene expression. (**a**) 853 up-regulated genes and 602 down-regulated genes (LumA ILC, n = 159 vs LumA IDC, n = 311, FDR < 0.05) in TCGA were validated in METABRIC (marked in red, the direction of the changes for DE genes were matched). (**b**) Proportion of immune phenotypes in LumA ILC (n = 157) and LumA IDC (n = 303). Tumors were classified into 6 immune-phenotypes (immune-phenotype 1–6) by Tamborero *et al*. and those in immune-phenotype 1–3 and 4–6 were defined as low immune tumors and high immune tumors, respectively. Chi-square test, ***p < 0.0005. (**c**) Expression of *CD274* (PD-L1), *PDCD1* (PD-1) and *CTLA4* in LumA ILC and LumA IDC of different immune phenotypes. High Immune LumA ILC and IDC have similar PDCD1, and CTLA4 expression as Basal and HER2 subtypes. Low immune (LumA ILC, n = 77, vs LumA IDC n = 221), high immune (LumA ILC, n = 80, vs LumA IDC n = 82), all (LumA ILC, n = 157 vs LumA IDC, n = 303). Two-way ANOVA for the effect of histological subtype on immune checkpoint gene expression, *p < 0.05, **p < 0.005, ***p < 0.0005. The effect of immune phenotype on immune checkpoint gene expression, p < 0.0005 for all genes. No significant interaction (p > 0.05) between histology and immune phenotype. (**d**) Proportion of high immune tumors in ILC subtypes (Proliferative n = 18, Reactive-like n = 34, Immune-related n = 40). Chi-square test for equality of proportions, ***p < 0.0005.

Upon overlapping DE genes between LumA ILC and LumA IDC (FDR < 0.05) from both datasets (TCGA and METABRIC), we identified 853 up-regulated and 602 down-regulated genes (Fig. 1a, Supplementary Table 3). Confirming prior studies^1,21^ and work from the recent TCGA ILC working group^2^, *CDH1* was the strongest downregulated gene in ILC as compared to IDC. Additionally, genes involved in extracellular matrix organization such as *MMP11* and *COL11A1* were also expressed at significantly lower levels in ILC. Conversely, *GDF9* - a TGF-β family member- and genes involved in fatty acid transport (*CD36, FABP4*) were up-regulated in LumA ILC. There was no significant association between the “top fold-change” genes (absolute log2 FC >2 in TCGA, or >1 in METABRIC) and survival in patients bearing LumA ILC (Supplementary Table 4).

To identify biological pathways that were significantly activated in LumA ILC compared to LumA IDC, we queried the induced DE genes in the MSigDB database^32^. Nine of the top 15 induced pathways were immune-related, including Response to Wounding, BioCarta IL17 Pathway, and BioCarta TCR Pathway (Supplementary Fig. S1, Supplementary Table 5). In order to investigate the immune pathway results further, we utilized data from a recent analysis of immune cell-type specific signatures across TCGA and the Genotype-Tissue Expression (GTEx) Project^33^. Briefly, Tamborero *et al*. used a gene set enrichment analysis method (GSVA)^34^ to identify immune cell populations in tumors and normal tissue samples, and then categorized all TCGA tumors with an immune phenotype score on a scale from 1–6, with 1 representing the lowest immune infiltrate, and 6 representing the highest immune infiltrate. We extracted this immune phenotype score for the LumA ILC and LumA IDC (Supplementary Fig. S2), and dichotomized those with immune-phenotype 1–3 as low-immune phenotypes and 4–6 as high-immune phenotypes. LumA ILC had a higher proportion of high-immune phenotypes (Fig. 1b) as compared to LumA IDC (53% vs 27%, Chi-square test p = 6.2e-7).

Analysis of the individual immune cell type signatures (Supplementary Table 6) from Tamborero *et al*.^33^, Davoli *et al*.^35^ and Li *et al*.^36^ showed that the majority (9/16, 7/8 and 5/6 respectively) of immune cell types were increased in LumA ILC compared with LumA IDC (Supplementary Figs S2, S3). In addition, we observed higher expression of *CD274* (PD-L1), *PDCD1* (PD-1) and *CTLA4* (Fig. 1c, Supplementary Table 3), which are the targets of FDA approved immune checkpoint inhibitors, and inhibition of these targets are currently being tested in breast cancer^37,38^. Other critical immune checkpoint genes such as *BTLA*, *IDO1*, *LAG3*, *TIGIT*, *HAVCR2* (TIM3) and *VSIR* (VISTA) were also highly expressed in LumA ILC (Supplementary Fig. S4). This was an important finding given recent studies showing that the expression levels of such genes are often correlated with the responsiveness of tumors to immunotherapies such as checkpoint blockade^39–41^, a promising line of therapy currently unexplored for patients with ILC. Ciriello *et al*. identified an *Immune-related* group of LumA ILC with activated immune involved pathways as compared to the other two groups (*Proliferative*, *Reactive-like*)^2^. Our data showed Immune-related LumA ILC also had the highest proportion of high immune phenotypes (Fig. 1d), which further confirmed the existence of a group of high immune tumors within ILC.

**Figure 2:**
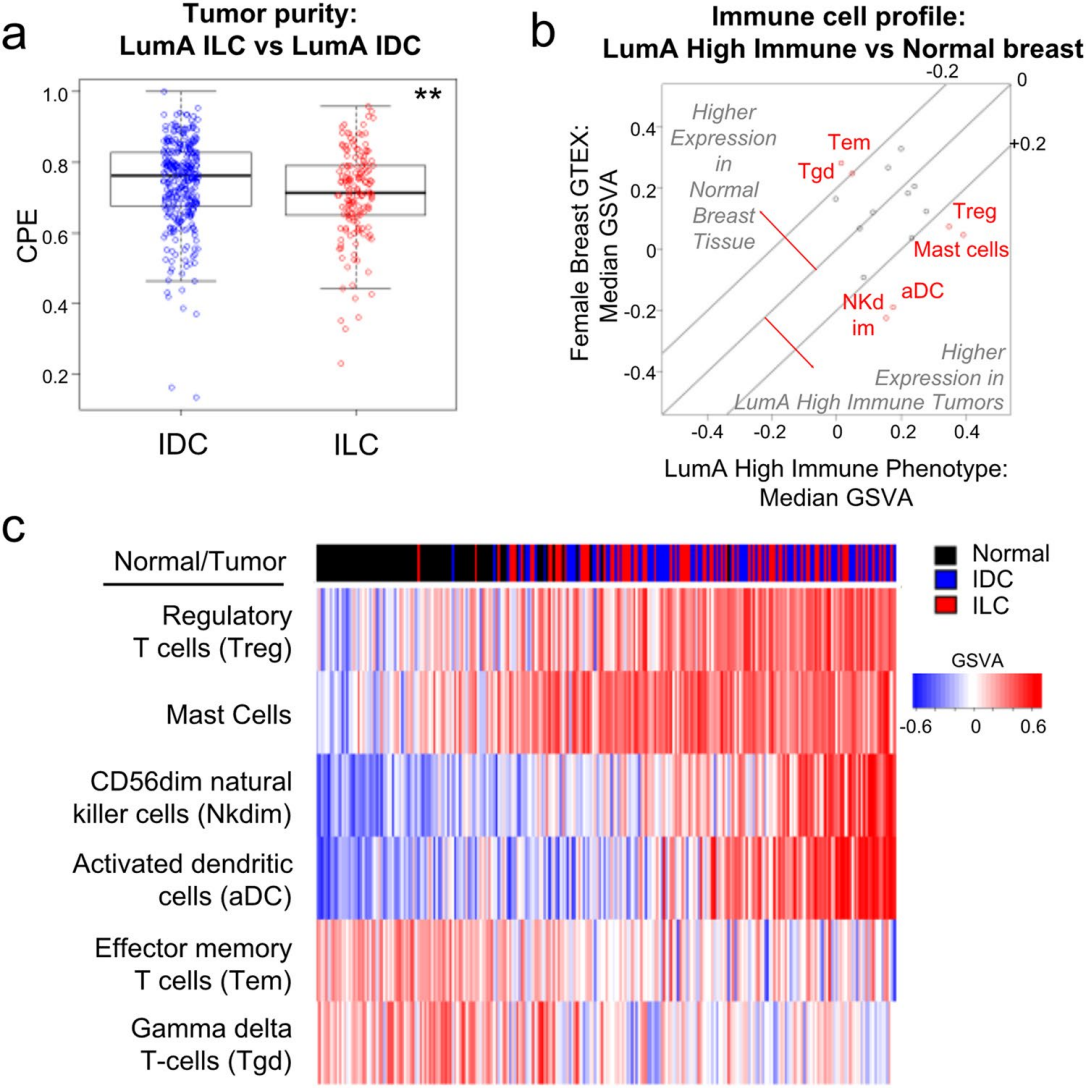
Immune signature difference is not a reflection of normal breast contamination. (**a**) Tumor purity score (CPE) of LumA ILC (n = 157) and LumA IDC (n = 307). Mann-Whitney U test, **p < 0.005. (**b**) LumA high immune tumors (n = 162) have different immune cell profile than normal female breast tissue (n = 90). Immune cell types with median GSVA difference >0.2 between normal breast tissue and LumA high immune tumors are marked in red: CD56dim Natural Killer cells (NK dim), activated dendritic cells (aDC), effector memory T-cells (Tem), gamma delta T-cells (Tgd). (**c**) Tumors and normal breast can be differentiated based on immune cell expression. Tumors/normal breast tissues in heatmap were sorted by sum (Treg+mast_cell + Nkdim + aDC) – sum(Tem, Tgd).

Given the unique growth properties of ILC, often growing as single line strands, we reasoned that this finding may be a result of the relatively sparse cellularity in ILC^21,42^. To test this, we compared the tumor purity scores of ILC and IDC samples using a Consensus measurement of Purity Estimations (CPE)^43^ that uses the median value of DNA, mRNA, methylation and/or IHC based scores (ESTIMATE, LUMP, ABSOLUTE, IHC). This analysis showed that LumA ILC does indeed have lower tumor purity compared to LumA IDC, consistent with previous findings^21,42^ (Fig. 2a). We next compared the immune cell profiles of LumA high immune tumors to normal breast tissues to check if the immune gene set differences are due to normal breast contamination. Again using the GSVA data for immune cell expression from Tamborero *et al*.^33^, we found LumA high immune tumors had higher expression of activated dendritic cells (aDC), mast cells, CD56dim natural killer cells (NK dim) and regulatory T-cells (Treg), and lower expression of effector memory T-cells (Tem) and gamma delta T-cells (Tgd) as compared to normal female breast tissues in GTEx (Fig. 2b). The high immune phenotype LumA tumors can be well separated from the normal breast tissues based on expression of these 6 immune cell types (Fig. 2c), suggesting the difference between LumA ILC and LumA IDC in immune signatures wasn’t a result of normal breast contamination. When comparing immune cell profiles between high immune LumA ILC and IDC, differences in CD56bright natural killer cells (NK bright), Tem cells, neutrophils, mast cells, and follicular helper T cells (Tfh) remained (Supplementary Figs S2, S3). Thus, ILC have a higher proportion of tumors with a high immune phenotype, and in addition, there are some qualitative differences in the types of immune cells that infiltrate the tumors.

To minimize a potential effect of different tumor cellularity on the identification of DE genes, we repeated the analysis after CPE correction, and identified 1360 genes differentially expressed in both TCGA and METABRIC datasets (Supplementary Fig. S5, Supplementary Table 3). Although some immune genes, like *IL7R*, and *CD36*, were now excluded from the list of DE genes, overall, the CPE correction resulted in changes in less than 10% of the DE genes (Supplementary Fig. S5). Furthermore, the immune pathways including Response to Wounding, Immunological Synapse, and BioCarta IL17 Pathway remained the dominant ILC-enriched pathways in the MSigDB pathway analysis (Supplementary Fig. S5), further indicating that these results were not a result of lower tumor purity. Collectively, the above data suggested higher immune infiltration in ILC compared to IDC.

### LumA ILC has lower protein translation efficiency than LumA IDC

**Figure 3:**
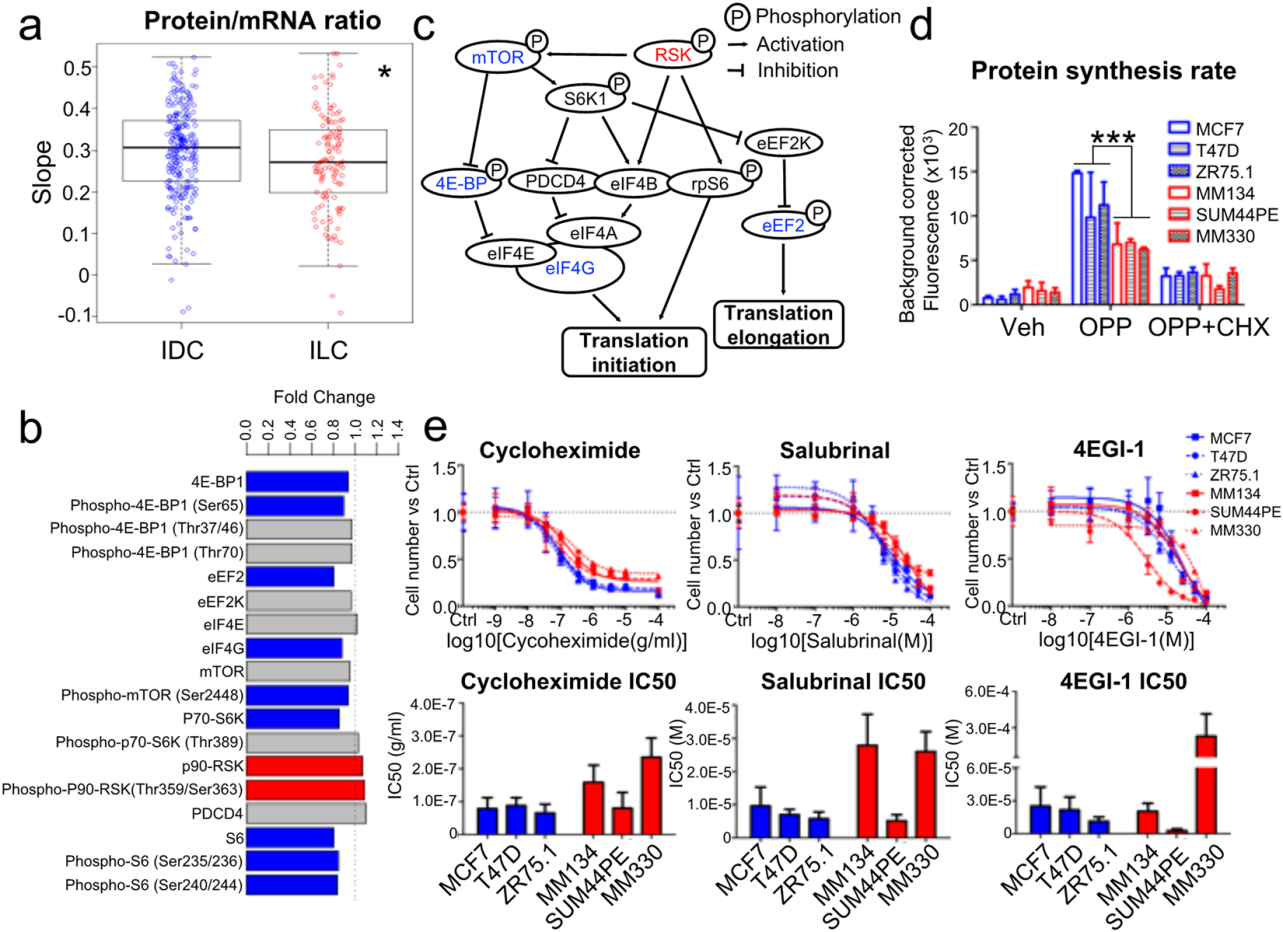
LumA ILC has lower protein translation efficiency than LumA IDC. (**a**) Protein/mRNA ratio in LumA ILC (n = 115) and LumA IDC (n = 246). Mann-Whitney U test, *p < 0.05. (**b**) Protein levels of translation regulators in LumA ILC vs LumA IDC. Protein expression data were from TCGA RPPA. Limma was used to compare the protein expression of LumA ILC to LumA IDC with CPE correction. Significant DE proteins (Benjamini-Hochberg method adjusted p-value < 0.05) were marked in red (up-regulated in LumA ILC) or blue (down-regulated in LumA ILC). (**c**) Regulation network of protein translation regulators in Fig. 2b. Modified from^65^. (**d**) Protein synthesis rate of ILC and IDC cell lines. O-propargyl-puromycin (OPP) labeled the newly synthesized proteins. Fluorescence representing the amount of OPP indicated the protein synthesis rate of cells. Cells without OPP labeling or pre-treated with cycloheximide (CHX) to inhibit protein synthesis served as negative controls. Representative data of two independent experiments were presented. Data are mean ± s.d. of 3 replicates. Two-way ANOVA, ***p < 0.001. (**e**) Dose response and IC50 of translation inhibitors in ILC and IDC cell lines. 4EGI-1 to inhibit the binding of eIF4E and eIF4G, cycloheximide to inhibit the tRNA translocation, salubrinal to inhibit eIF-2α were used. Representative data of at least two independent experiments were presented. Data in dose response curves are mean ± s.d. of 6 replicates. Data in bar graphs of IC50 are mean + upper limit of 95% confidence intervals. Two-tailed t-test was performed to compare the IC50s between ILC and IDC cell lines. The p-values for cycloheximide, salubrinal and 4EGI-1 are 0.15, 0.17 and 0.42, respectively.

Other pathways that were significantly increased in ILC compared to IDC included Reactome Peptide Chain Elongation and Ribosome (Supplementary Fig. S5, and Supplementary Table 7), prompting us to investigate whether there are differences in the rate of protein synthesis between ILC and IDC. To test this, we first compared the ratios of total RNA to total protein in LumA ILC vs LumA IDC by extracting the expression levels of 156 proteins from the TCGA RPPA data set (excluding phosphoproteins), and determining correlation with their respective mRNA expression using a linear regression model. LumA ILC samples showed significantly less steep slopes than LumA IDC, reflecting a lower protein/mRNA ratio in LumA ILC (Fig. 3a). Next we applied the same methodology to the mass spectrometry data from The Clinical Proteomic Tumor Analysis Consortium (9117 selected proteins, LumA ILC n = 8, LumA IDC n = 16)^44^, which confirmed the lower protein/mRNA ratio in LumA ILC (Supplementary Fig. S6). Furthermore, analysis of key regulators of protein translation initiation and elongation also revealed lower expression in ILC compared to IDC including eIF4G, phospho-4E-BP1 (Ser65), eEF2, ribosome protein S6 (S6), phospho-S6 (Ser235/236, Ser240/244), p70-S6K and phospho-mTOR (Ser2448) (Fig. 3b), in agreement with the lower protein translation rates in LumA ILC compared to LumA IDC (Fig. 3c). Therefore, we reason that, while expression of critical translation initiation factors is decreased, there is a higher expression of ribosomal proteins in LumA ILC, likely as a compensatory mechanism, mimicking what has been recently described in melanoma samples with low rates of protein translation^45^.

To more directly assess the differences in protein translation in ILC and IDC, we measured protein synthesis rates in three ILC (MDA-MB-134VI, SUM44PE, and MDA-MB-330) and three IDC (MCF7, T47D, and ZR75.1) cell lines. With O-propargyl-puromycin (OPP) - a structural analog of aminoacyl-tRNA - labelling the newly synthesized protein, this analysis showed significantly lower protein synthesis rates in the ILC as compared to IDC cell lines (Fig. 3d), in agreement with the data from the clinical samples. Finally, we tested the effects of the protein translation inhibitors cycloheximide, 4EGI-1 and salubrinal, and detected a trend towards resistance to protein translation inhibitors in the ILC lines, especially in MDA-MB-134VI and MDA-MB-330 (Fig. 3e). Collectively, these data indicate that protein translation rates are lower in LumA ILC compared to LumA IDC.

### LumA ILC is more bioenergetically quiescent than LumA IDC

**Figure 4:**
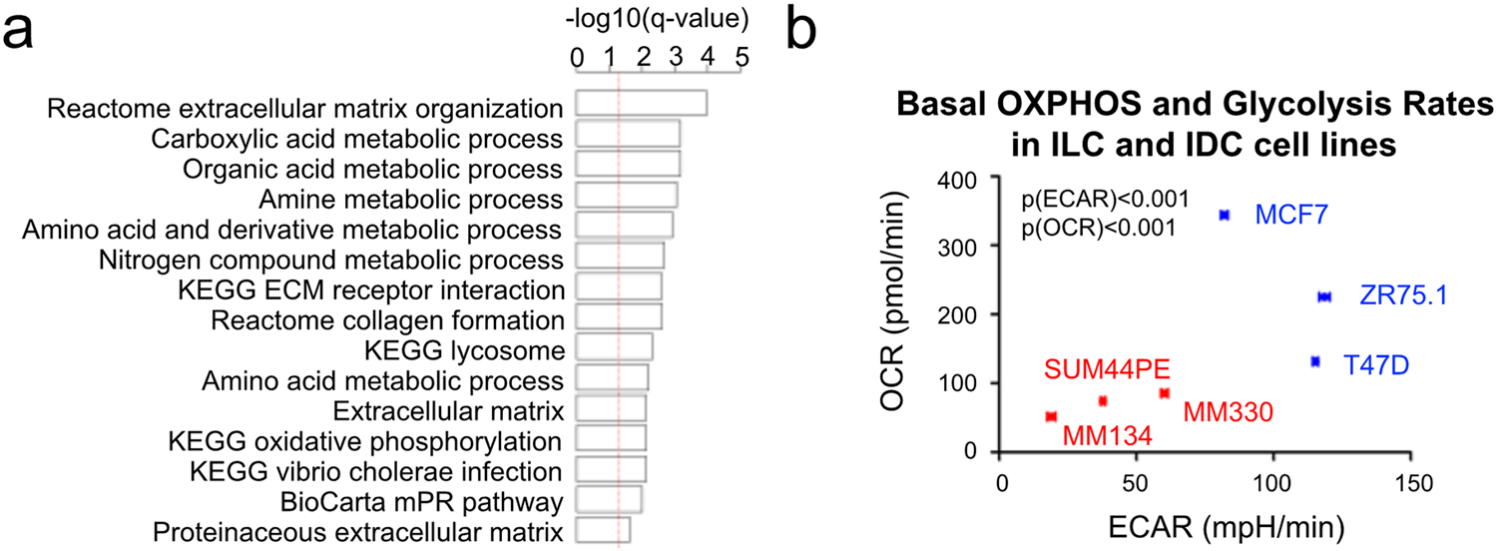
LumA ILC is more bioenergetically quiescent as compared to LumA IDC. (**a**) Top 15 inhibited pathway in LumA ILC compared to LumA IDC. –log10(0.05) is marked with red line. (**b**) The basal OXPHOS and glycolysis rate of ILC and IDC cell lines. Oxygen consumption rate (OCR) and extracellular acidification rate (ECAR), which are indicators of OXPHOS and glycolysis rates respectively were measured with Seahorse XF^e^96 analyzer. Representative data of two independent experiments were presented. Data are mean ± SEM of 3 repeated measurements. Each measurement measured 6 or 8 biological replicates. Two-way ANOVA, p-values for ECAR[p(ECAR)] and OCR[p(OCR)] between ILC and IDC cell lines were calculated independently.

The third set of pathways that were significantly different between LumA ILC and IDC were related to metabolism, including Carboxylic Acid Metabolic Process, Amino Acid Metabolic Process, and Oxidative Phosphorylation (Supplementary Fig. S1). These pathways remained significantly lower in ILC after CPE correction (Fig. 4a). To assess potential differences in metabolism between ILC and IDC, we measured the basal oxygen consumption rate (OCR) and the basal extracellular acidification rate (ECAR) as indicators of the oxidative phosphorylation (OXPHOS) and glycolysis, respectively, in cell line models. This analysis revealed that all three ILC cell lines (MDA-MB-134VI, SUM44PE, MDA-MB-330) had lower OCR and ECAR rates compared to the IDC cell lines (MCF-7, T47D, and ZR-75.1) (Fig. 4b). These findings support *in-silico* analysis of the TCGA data, suggesting that LumA ILC is characterized by lower rates of cellular metabolism.

## Discussion

ILC is a histological subtype of breast cancer with unique clinical and molecular features that remains largely unexplored. Recent sequencing and molecular profiling studies have begun to uncover biologically important pathways mediating the progression of this understudied disease^2,27,29^. While mutation and copy-number data have been used to compare ILC and IDC, and gene expression data has been used to classify ILC into distinct molecular subtypes, detailed comparison of ILC and IDC at the mRNA and protein levels has not been addressed. Such a transcriptional comparison could be highly informative and uncover novel therapeutic targets, given the recent finding that cancer dependencies can be best predicted by RNA expression levels as opposed to DNA mutation and copy number^46^. In this study, we addressed this critical need and uncovered several pathways enriched and depleted in ILC versus IDC, which might help explain some clinical features of ILC and also hint at novel therapeutic options.

Recently, Fu *et al*. combined gene expression data from six breast cancer cohorts, and built a 46-gene signature to classify ILC and IDC using shrunken centroid and elastic net approaches^47^. However, the relative high misclassification rate (~40%) weakened the robustness of their pathway analysis. Furthermore, the effects of breast cancer intrinsic subtypes and differences in tumor purity on gene expression were not taken into account^47^. In contrast, in this study, we limited our analyses to LumA ILC and LumA IDC. In addition, we performed tumor purity-corrected DE analysis to reduce potential confounding influence of non-tumor cells within tumors. This is critical as ILC is known to have lower levels of tumor cellularity. CPE correction uncovered additional pathways such as those related to protein translation. The majority of immune signatures persisted after CPE correction indicating the robustness of our differential immune activity finding in LumA ILC vs LumA IDC. A limitation of our study is our inability to validate the CPE-corrected TCGA DE genes in the METABRIC data, which lack CPE score information. Nevertheless, using both datasets for initial analysis enhanced the strength and stringency of our DE gene calling, allowing us to focus on biologically important pathways.

*Immune-related* ILC was an ILC subgroup that was identified in the analyses of both the TCGA and RATHER consortia. Compared to the two other ILC subtypes (*Proliferative*, *Reactive-like*), *Immune-related* ILC in TCGA had higher expression of interleukins, chemokine receptors and ligands, and also increased macrophage-associated signaling^2^. The RATHER consortium showed that chemokines, cytokines and innate immune signaling were enriched in their immune-related subtype^27^. In our analysis, we found that LumA ILC had up-regulated immune signatures as compared to LumA IDC. Comparison of immune cell profiles between normal breast tissues and LumA ILC/IDC also suggested that the high immune signature in LumA ILC is not caused by lower tumor cellularity. However, our analysis of immune signatures is based on the expression of immune genes, and caution should be taken when correlating gene expression and actual quantities of immune cells. Future studies should apply additional methodologies such as IHC, FACS, or single-cell sequencing to confirm and expand our findings, and also to identify which cells are expressing immune-related genes. A recent study by Desmedt *et al*.^48,49^ showed that on average lymphocytic infiltration was lower in ILC compared to IDC, but also supported the idea that there was a subset of ILCs (15%) with high immune infiltration, and that there were differences in immune composition.

Immunotherapy and its integration with conventional and novel targeted cancer therapy provide new opportunities for breast cancer. The infiltration of lymphocytes has been shown to be a favorable prognostic factor and to predict response to neoadjuvant chemotherapy^50–52^. Currently, studies investigating the association between prognosis and lymphocyte infiltration in ILC are very limited. Engels *et al*. showed that the high immune-susceptible group which is characterized by extensive infiltration of CD8+ T cells and NK cells, had significantly longer relapse-free period than the low immune-susceptible group in IDC but not in ILC, a result that could be attributable to the small sample size of ILC (n = 66) in that study^53^. Incongruent results were also reported for the association between lymphocyte infiltration and survival in ER positive breast cancers^54–56^. Desmedt *et al*.^48^ also described limited prognostic value of TIL, but further analyses of spatial distributions of immune cells as recently described by Heindl *et al*.^57^ is warranted. Furthermore, recent studies indicated that cancers with PD-L1 overexpression had better response to anti-PD-1 therapy^58^. The higher expression of *PDCD1* (PD-1) and *CD274* (PD-L1) in LumA ILC suggests that anti-PD-1 therapy may be more effective in LumA ILC than LumA IDC. Of note, there is a wide range of expression of many of the checkpoint genes within LumA ILC, and further studies are required to understand what differentiates tumors with high vs low PD-1 and PD-L1 expression.

Gene expression is regulated by transcription, translation, and turnover of protein and mRNA. The lower protein/mRNA ratio can be attributed to higher mRNA levels via increased transcription and decreased mRNA degradation rates, and/or lower protein levels via decreased translation and increased protein degradation rates. Here, we mainly focused on protein translation, as the other processes were not significantly changed based on our pathway analysis. It is well known that dysregulation of protein translation is involved in the development and progression of various tumor types^59–62^. In breast cancer, high levels of eIF4E and phosphorylation of 4E-BP1 and S6 are correlated with worse survival^63–65^. Our study demonstrated that LumA ILC has down-regulated protein synthesis compared to LumA IDC. In support of this data, ILC cell lines were less responsive to protein synthesis inhibitors compared to IDC cell line. These data may prompt retrospective analysis of prior clinical trials using inhibitors of protein translation in a histological subtype-dependent manner. In addition, these data support further pre-clinical analysis to evaluate whether ILC and IDC differ in response to treatments that target translation (e.g. MTOR inhibitors).

Finally, we discovered that multiple metabolism-related pathways including OXPHOS and glycolysis were down-regulated in LumA ILC. Recent studies demonstrated that ILC showed lower uptake of ^18^F-Fluorodeoxyglucose than IDC on PET-CT, an indicator of glucose metabolism^66–68^. Few studies thus far have assessed the difference in OXPHOS between ILC and IDC. Kim *et al*. proposed that many ILCs belonged to the mitochondrial metabolic subtype, however, this was based solely on expression of ATP synthase, SDHA or SDHB^69^. Clearly, more studies need to be performed, with an obvious open question being whether the observed low bioenergetics is the cause or the result of the lower proliferation rates of ILC^7,70^. And while a similar cause-effect question applies to the observed differences in protein synthesis, it is reasonable to propose that the lower overall metabolism, protein synthesis, and cell proliferation are associated with described lower response rates to chemotherapy.

The PI3K/AKT/mTOR pathway plays a central role in cell growth, metabolism, and protein translation. mTORC1 controls mitochondrial biogenesis and the transcription of genes encoding proteins involved in OXPHOS^71–73^, which is in agreement with our finding of lower mTOR activity and decreased expression of multiple OXPHOS genes in LumA ILC. Another major role of mTOR is to activate translation initiation and elongation through the phosphorylation of 4E-BP and S6K1^60,74^, which we also found to be less activated in ILC. The lower mTOR activity and signaling is surprising given increased activity of AKT and PI3K in LumA ILC compared to LumA IDC^2^ (Supplementary Table 8), suggesting that ILCs may have distinct mechanisms regulating mTOR activity.

ILC exhibits later recurrences than IDC^10–13^, and has been described to have more micrometastatic disease^75^. While the increased rates of late recurrence may simply be a reflection of the slower proliferation rates, it may also be explained by tumor dormancy^76^, often associated with growth arrest, persistence within the microenvironment, and therapeutic resistance^77^. Immune surveillance, microenvironmental milieu including extracellular matrix and stromal cells, and angiogenesis are critical for cell to enter and maintain the dormancy state^77^. The low glucose metabolism, and low rates of protein synthesis, coupled with a more active immune response in LumA ILC, might create a permissive environment for tumor dormancy, causing late recurrences in some patients.

In conclusion, our analyses revealed that LumA ILC had up-regulated immune response, down-regulated protein translation rate, and were more bioenergetically quiescent than LumA IDC. We believe that our findings provide the molecular foundation to further explore several unique clinical characteristics of ILC, ultimately leading to improved prevention, diagnosis and treatment of this understudied subtype of breast cancer.

## Methods

### Cell culture and reagents

MCF7 and MDA-MB-330 (MM330) (American Type Culture Collection [ATCC], Manassas, VA, USA) were cultured in DMEM (11965; Life Technologies, Carlsbad, CA, USA) +10%FBS (26140; Life Technologies). T47D (ATCC) and ZR75.1 (ATCC) were cultured in RPMI 1640 (11875; Life Technologies) +10%FBS. MDA-MB-134VI (MM134) (ATCC) and SUM44PE (Asterand Bioscience, Detroit, MI, USA) were maintained as described previously^78^. All lines were incubated at 37 °C in 5% CO_2_.

Cycloheximide (C4859; Sigma-Aldrich, St. Louis, MO, USA), 4EGI-1 (S7369; Selleck Chemicals, Houston, TX, USA), and Salubrinal (SC-202332A; Santa Cruz, Dallas, TX, USA) were dissolved in DMSO (4-X; ATCC).

### Protein synthesis and cell proliferation assay

To measure protein synthesis rates, we used a Protein Synthesis Assay Kit from Cayman Chemical (Ann Arbor, MI, USA. Cat No. 60110). 50 K/well cells were seeded in 96 well plates. Cells were treated with O-propargyl-puromycin (OPP) and cycloheximide following manufacturer’s instructions. Cells were then fixed and stained with 5 FAM-Azide. Fluorescence (excitation/emission = 485/535 nm) was measured using the VICTOR X4 plate reader (PerkinElmer, Waltham, MA, USA).

Cell proliferation were quantified using the Fluoreporter double-stranded DNA quantification kit (F2692; Life Technology) following manufacturer’s instructions.

### Analysis of OXPHOS and glycolysis

Seahorse XF96 Analyzer (Seahorse Bioscience, Billerica, MA, USA) was used to analyze the oxygen consumption rate (OCR) and extracellular acidification rate (ECAR). 96-well Seahorse tissue culturing plates were pre-incubated with Cell-Tak Cell and Tissue Adhesive (354240; Corning, Corning, NY, USA). Cells were seeded in unbuffered DMEM media at a density of 80 K per well. Cells were incubated for 1 hour without CO_2_ at 37 °C. OCR and ECAR rates were measured as previous described by us^79,80^. The basal OCR and ECAR rates were measured 3 times without adding any inhibitors.

### Identification of differentially expressed genes

Gene expression data from The Cancer Genome Atlas (TCGA) and Molecular Taxonomy of Breast Cancer International Consortium (METABRIC) were downloaded from the Gene expression Omnibus database [GEO: GSE62944] and Synapse software platform (syn1688369; Sage Bionetworks, Seattle, WA, USA) respectively.

TCGA tumors were assigned to one of the five intrinsic subtypes based on PAM50 similarly as described in Curtis 2012^29^. Briefly, we first created an ER balanced sub-samples by combining all ER- tumors (N = 174) and the same number of ER+ tumors randomly drawn from TCGA. Log2 transcripts per million (TPM) of all tumors were then median centered by extracting the median calculated from ER balanced sub-samples. Genefu R package^81^ was used to assign intrinsic subtypes for all tumors using median centered data. We repeated drawing ER balanced samples 100 times, and the most frequently assigned subtypes were use as final subtypes.

Raw gene expression counts from TCGA, and R package DESeq2^30^ was used to analyze differentially expressed gene in LumA ILC and LumA IDC. Consensus measurement of Purity Estimations (CPE) developed by Aran *et al*.^43^, and histology groups were inputted as parameters in the DESeq2 design formula/matrix, called “CPE correction”. For CPE uncorrected DE analysis, only histology groups were inputted into DESeq2. In microarray data from METABRIC, probes with the highest interquartile range were selected for genes that matched to multiple probes. Significance Analysis of Microarrays (SAM) was used to detect the DE genes with METABRIC data. FDR <0.05 were used to call DE genes, and TCGA DE genes were validated in METABRIC (LumA ILC: n = 65; LumA IDC: n = 533) using the same cutoff.

Since the number of TCGA cases decreased slightly (due to unavailability of CPE score for 2 LumA ILC and 4 LumA IDC), we repeated the DE analysis with altered numbers (LumA ILC: n = 157; LumA IDC: n = 307). This analysis confirmed that changes in DE genes and pathways were not caused by change in numbers of tumor samples (Supplementary Fig. S5). In figures with the GSVA or immune phenotype data from Tamborero *et al*. (Figs 1b–d, 2b,c, Supplementary Figs S2, S3a, S4), all of their 924 tumors were used (LumA ILC: n = 157; LumA IDC: n = 303). All other analyses with TCGA used the complete set of tumors (LumA ILC: n = 159; LumA IDC: n = 311).

Reverse phase protein array (RPPA) data of TCGA tumors were downloaded from The Cancer Proteome Atlas (Level 4 data, data release version 4.0. MD Anderson Cancer Center, Houston, TX). R package Limma^82^ was used to perform CPE corrected differentially expressed protein analysis with CPE and histology groups (LumA ILC: n = 113; LumA IDC: n = 242) as parameters in the design formula/matrix. Full list of differentially expressed proteins is available in Supplementary Table 9.

### Pathway analysis

DE genes consistently up- or down-regulated in TCGA and METABRIC were used in pathway analyses. 2531 pathways, which were contributed by BioCarta, GO, KEGG, Reactome, containing 5–2000 genes, were obtained from Molecular Signature Database (MSigDB Version 5.1. Broad Institute, Cambridge, MA, USA). Fisher’s exact test was used to determine significantly enriched pathways, using FDR <0.05 as cutoff.

Gene Set Enrichment Analysis (GSEA Version 2.2.2. Broad Institute) was also conducted with full DE gene list as a validation. Default settings in *GseaPreranked* were used except the following parameters: “Enrichment statistic” was “Classic”; “Min size: exclude smaller sets” was set to be 0.

### Survival analysis in METABRIC

Survival analysis was performed with METABRIC data on METABRIC validated DE genes with absolute log2FoldChange >1 in METABRIC or absolute log2FoldChange >2 in TCGA. LumA ILC (n = 65) or LumA IDC (n = 533) tumors were split into two groups by median gene expression of LumA ILC or LumA IDC, respectively. Log-rank test was conducted to compare the survival distribution of the two groups. Log-rank p-value was further corrected with Benjamini–Hochberg methods.

### Estimation of abundance of immune cell population and classification of immune-phenotypes

GSVA scores for each immune cell type signature across TCGA tumors were downloaded from Tamborero *et al*. using their pan-cancer normalization and across GTEx tissues using their pan-site normalization^33^. Similar to their approach, a median GSVA score >0.2 was used as the cutoff for different immune cell abundance between two groups. The immune-phenotype classification of LumA tumors was also downloaded directly from Tamborero *et al*.^33^.

Immune cell signatures defined as recently described in Davoli *et al*.^35^ are available in Supplementary Table 6. For each gene, the TCGA log2TPM data (without CPE correction) were normalized by mean and standard deviation. The average gene expression of each signature was then calculated in LumA ILC and in LumA IDC.

### Protein/mRNA ratio

Phosphorylated or cleaved proteins representing active but not total protein levels were excluded from RPPA, resulting in available expression data for 156 proteins. The mRNA expression levels from TCGA (in units of log2TPM) were median centered for each gene, and linear regression was fitted with corresponding RPPA protein expression data. Slope of the linear regressions were calculated, representing protein/mRNA ratios in individual tumors. List of the 156 proteins is available in Supplementary Table 10.

### Data availability

MRNA expression data from TCGA and METABRIC, and RPPA data of TCGA tumors are available as indicated above. Other datasets generated and/or analyzed during this study are included in this published article and its Supplementary Information files. R codes used in the current study are available from the corresponding author on request.

## Electronic supplementary material

Supplementary information

Supplementary table 2

Supplementary table 3

Supplementary table 5

Supplementary table 6

Supplementary table 9

Supplementary table 10
