# Supplementary material for: Invasive lobular and ductal breast carcinoma differ in immune response, protein translation efficiency and metabolism
Source: Sci Rep. 2018 May 8;8:7205. doi: 10.1038/s41598-018-25357-0 (PMC5940770; doi:10.1038/s41598-018-25357-0)
Supplement: Supplementary file 1 — Supplementary information [file 41598_2018_25357_MOESM1_ESM.pdf]

Supplementary figures and tables for:

Invasive lobular and ductal breast carcinoma differ in immune response, protein translation efficiency and metabolism

Tian Du[1,2], Li Zhu[3], Kevin M. Levine[1,4], Nilgun Tasdemir[1,5], Adrian V Lee[1,5], Dario AA Vignali[6], Bennett Van Houten[5], George C. Tseng[3,7], Steffi Oesterreich[1,5]*

1. Womens Cancer Research Center, UPMC Hillman Cancer Center, Magee Womens Research Institute, Pittsburgh, PA 15213, USA
2. School of Medicine, Tsinghua University, Beijing 100084, China
3. Department of Biostatistics, University of Pittsburgh, Pittsburgh, PA 15213, USA
4. Department of Pathology, University of Pittsburgh, Pittsburgh, PA 15213, USA
5. Department of Pharmacology & Chemical Biology, University of Pittsburgh, Pittsburgh, PA 15213, USA
6. Department of Immunology, University of Pittsburgh School of Medicine, Pittsburgh, PA 15213. Tumor Microenvironment Center, UPMC Hillman Cancer Center, Pittsburgh PA 15232, USA
7. Department of Computational & Systems Biology, Pittsburgh, PA 15213, USA

Supplementary Figures

Fig. S1

Differentially expressed pathways between LumA ILC and LumA IDC

(a)(b) Top 15 activated (a) or inhibited (b) pathways in ILC (ranked by adjusted p-value). Fisher's exact test was performed on the 853 up-regulated and 602 down-regulated DE genes in Fig. 1a. –log10(0.05) is marked with red line. Gene set enrichment analysis (GSEA) were also conducted as a validation of fisher's exact test (Supplementary table 5).

Fig. S2

LumA ILCs have higher immune phenotypes because of higher GSVA scores for nearly all cell types

(a) Increased proportion of higher immune phenotype class for LumA ILC. LumA ILC n=157, LumA IDC n=303. Tumors were classified into 6 immune-phenotypes (immune-phenotype 1-6) using hierarchical agglomerative clustering by Tamborero et al. [33]. Immune infiltration and cytotoxic component increased from immune-phenotype 1 to immune-phenotype 6. (b) LumA ILC and LumA IDC have similar GSVA score for all immune cell types, but 13/16 are higher in ILC vs IDC. Lines for a median GSVA difference>0.2 are shown. (c) Immune cell type comparison between high immune LumA ILC vs high immune LumA IDC. Immune cell signatures were from Tamborero et al. [33]. Mann-Whitney U tests were performed, and q-values represent multiple comparisons correction using Benjamini-Hochberg method.

Fig. S3

Immune expression was increased in ILC vs. IDC for the majority of immune cell types using 3 different gene signatures for immune cell types.

(a) Immune cell type expression from Tamborero et al. [33]. (b) Immune cell type expression from Davoli et al. [35]. (c) Infiltration score for specific immune cell lineages from Li et al. [36]. Immune cell infiltration scores for each patient were

calculated in Li et al. [36] and directly used in our study. For a, b, and c, Mann-Whitney U tests were performed, and q-values represent multiple comparisons correction using Benjamini-Hochberg method.

Fig. S4

Expression of immune-checkpoint genes in LumA ILC (n=157) and LumA IDC (n=303).

Gene expression data were obtained from TCGA without CPE correction. Two-way anova for the effect of histological subtype on immune checkpoint gene expression, *$p<0.05$, **$p<0.005$, ***$p<0.0005$. The effect of immune phenotype on immune checkpoint gene expression, $p<0.0005$ for all genes. No significant interaction ($p>0.05$) between histology and immune phenotype.

Fig. S5

Differentially expressed genes and pathways with CPE correction.

(a) With tumor purity (CPE) correction, 789 and 571 genes (marked in red) were up- and down-regulated in both TCGA and METABRIC (LumA ILCs, n=157 vs LumA IDCs, n=307, p-value<0.05). (b) DE gene number changes after CPE correction. Blue: CPE not corrected (LumA ILC, n=159; LumA IDC, n=311); Red: CPE corrected (LumA ILC, n=157; LumA IDC, n=307). (c) DE gene number changes influenced by the change of the number of patients. Blue: LumA ILC, n=159 vs LumA IDC, n=311; Red: LumA ILC, n=157 vs LumA IDC, n=307. The sets of patients were identical to the sets in Fig. S2b. All DE analyses were performed without CPE correction. (d) Top 15 activated pathways in LumA ILCs with CPE correction. –log10(0.05) is marked with red line.

Fig. S6

LumA ILC has lower protein/mRNA ratio than LumA IDC.

Slope of linear regression in LumA ILC (n=8) and LumA IDC (n=16) using TCGA mass spectrometry data. For each patient, slope of linear regression was calculated with protein expression level (log2 iTRAQ ratios) as y-axis and TCGA mRNA level (log2TPM) as x-axis. Protein expression data were obtained from Mertins 2015. We selected 9117 protein/genes based on data availability from

the gene list used by Mertins et al [44] in their calculation of protein-mRNA correlations.

Supplementary tables

Supplementary table 1

Patient summary in TCGA, METABRIC, and Ciriello et al, Cell 2015 datasets.

Supplementary table 2

TCGA tumor histology and PAM50 assignment

Supplementary table 3

METABRIC validated TCGA DE genes before and after CPE correction

Supplementary table 4

Adjusted p-value of selected DE genes in survival analysis

Supplementary table 5

Pathway analysis before and after CPE correction (Fisher's exact test and GSEA)

Supplementary table 6

Genes in immune cell signatures

Supplementary table 7

METABRIC validated TCGA DE genes in protein translation/regulation pathways.

Supplementary table 8

Alteration of proteins in PI3K/AKT/mTOR pathway (CPE corrected).

Supplementary table 9

DE proteins in LumA ILC vs LumA IDC and ER+ ILC vs ER+ IDC (CPE corrected)

Supplementary table 10

The list of 156 proteins used in the calculation of protein/mRNA ratio

Figure S1: Differentially expressed pathways between LumA ILC and LumA IDC

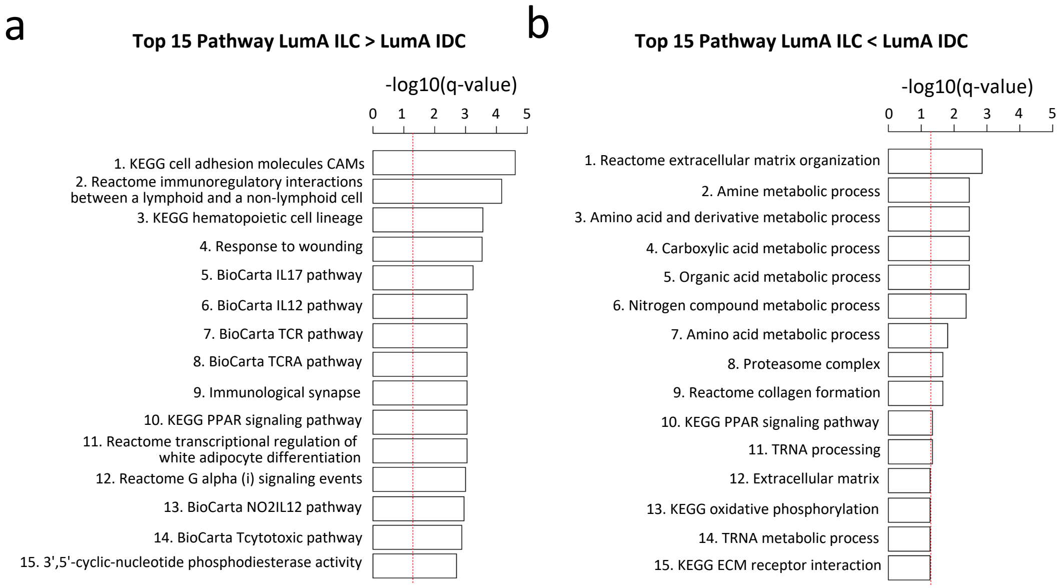

Figure S2: LumA ILCs have higher immune phenotypes because of higher GSVA scores for nearly all cell types

a

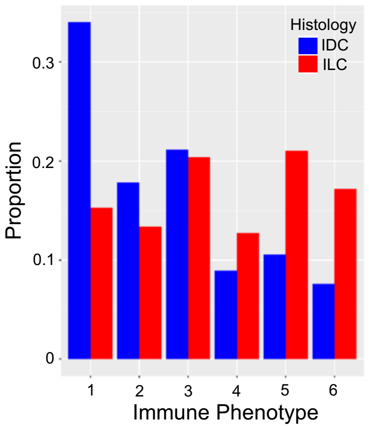

b

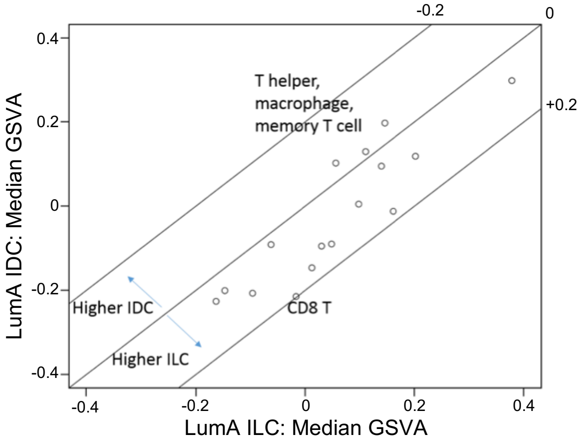

c

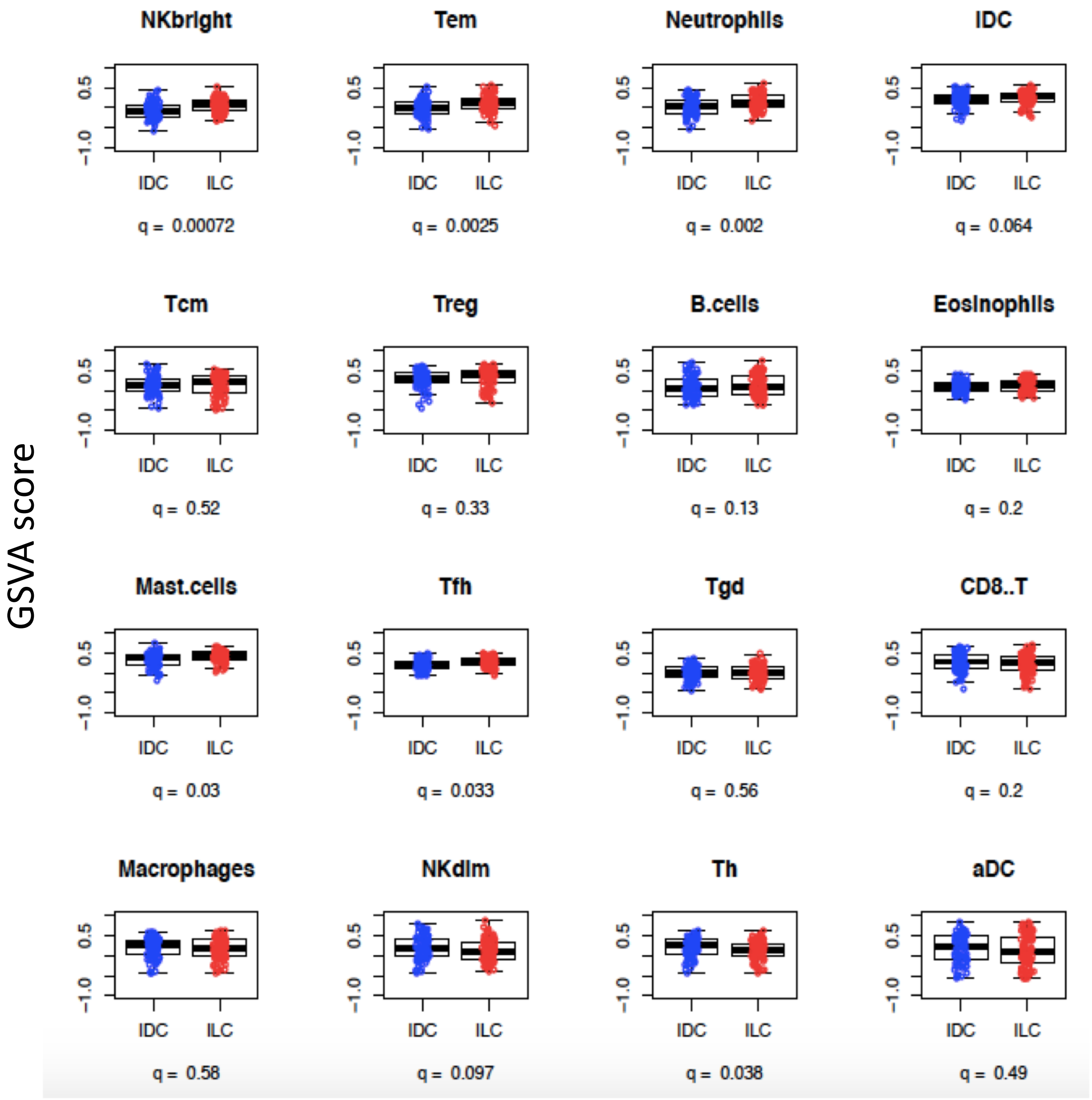
GSVA score
NKbright
q = 0.00072
Tem
q = 0.0025
Neutrophils
q = 0.002
IDC
q = 0.064
Tcm
q = 0.52
Treg
q = 0.33
B.cells
q = 0.13
Eosinophils
q = 0.2
Mast.cells
q = 0.03
Tfh
q = 0.033
Tgd
q = 0.56
CD8..T
q = 0.2
Macrophages
q = 0.58
NKdim
q = 0.097
Th
q = 0.038
aDC
q = 0.49
IDC
ILC
0.5
-1.0

Figure S3: Immune expression was increased in ILC vs. IDC for the majority of immune cell types using 3 different gene signatures for immune cell types.

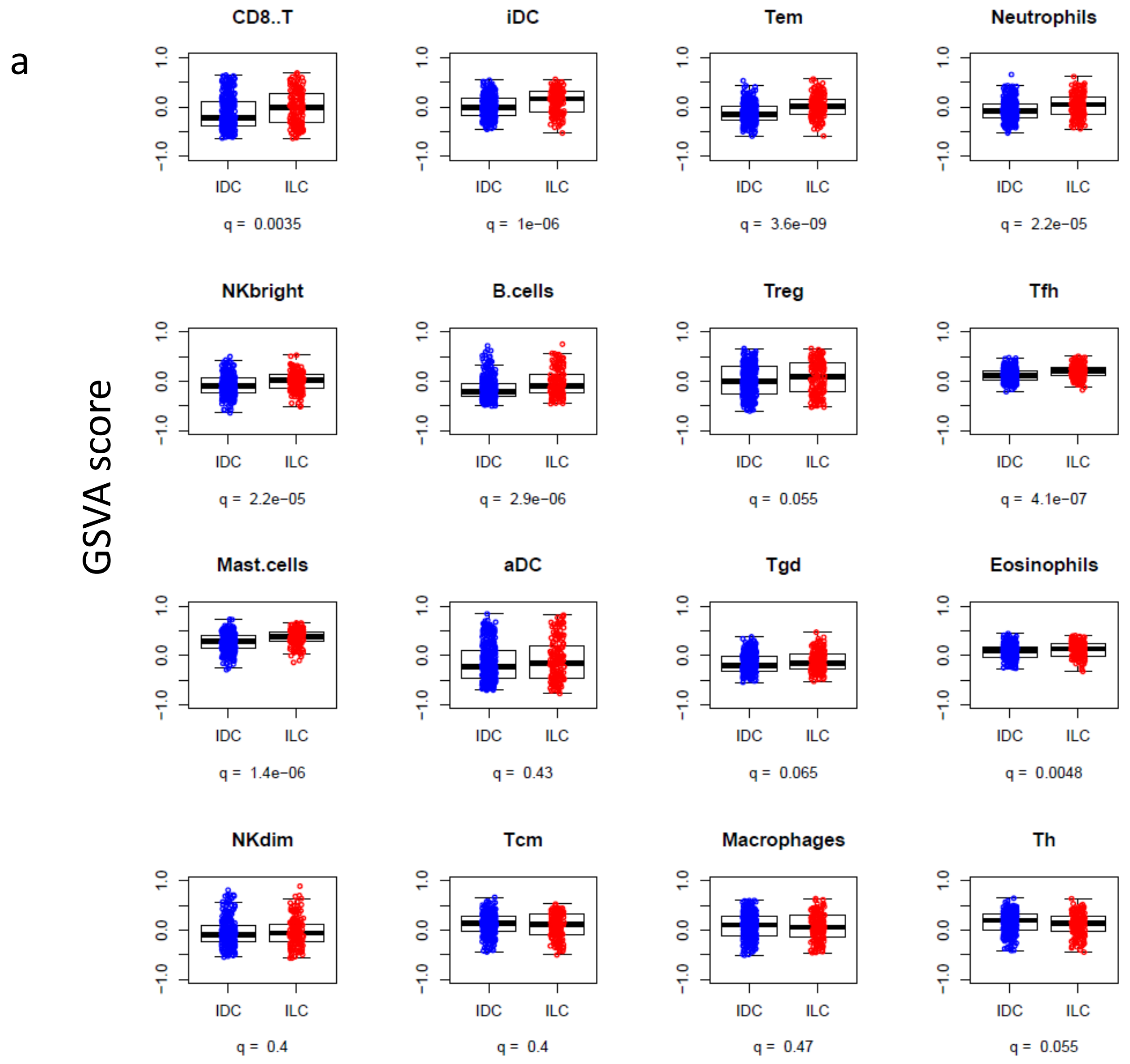

b

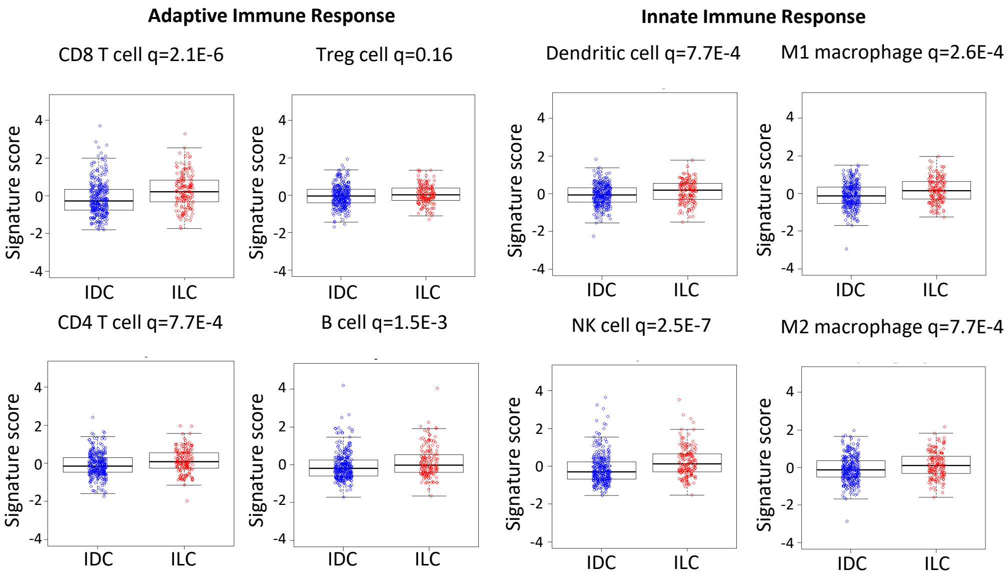

Adaptive Immune Response
Innate Immune Response
CD8 T cell q=2.1E-6
Treg cell q=0.16
Dendritic cell q=7.7E-4
M1 macrophage q=2.6E-4
CD4 T cell q=7.7E-4
B cell q=1.5E-3
NK cell q=2.5E-7
M2 macrophage q=7.7E-4
Signature score
4
2
0
-2
-4
IDC
ILC

c

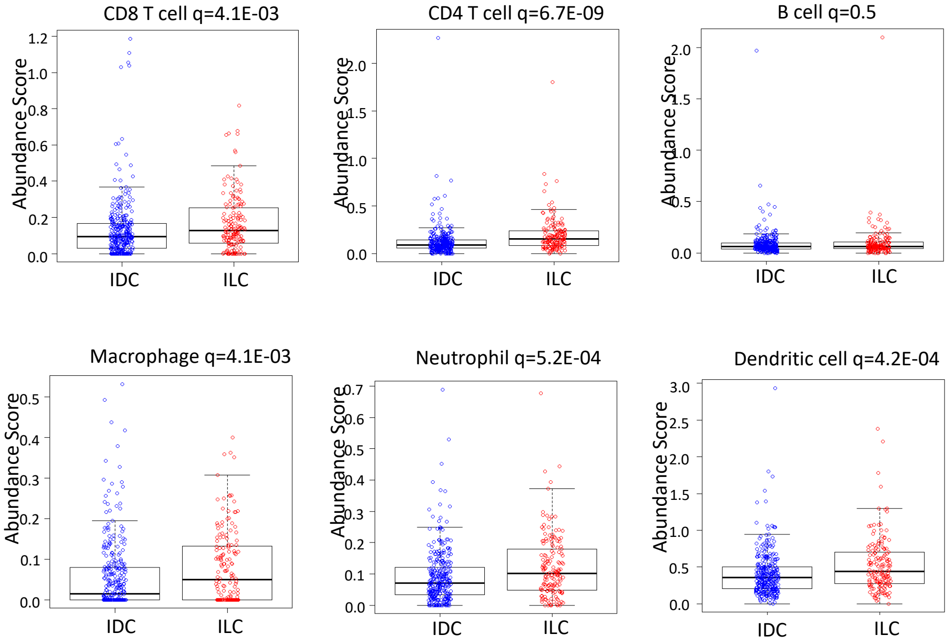

CD8 T cell q=4.1E-03
CD4 T cell q=6.7E-09
B cell q=0.5
Macrophage q=4.1E-03
Neutrophil q=5.2E-04
Dendritic cell q=4.2E-04
Abundance Score
IDC
ILC

Figure S4: Expression of additional immune-checkpoint genes in LumA ILC (n=157) and LumA IDC (n=303).

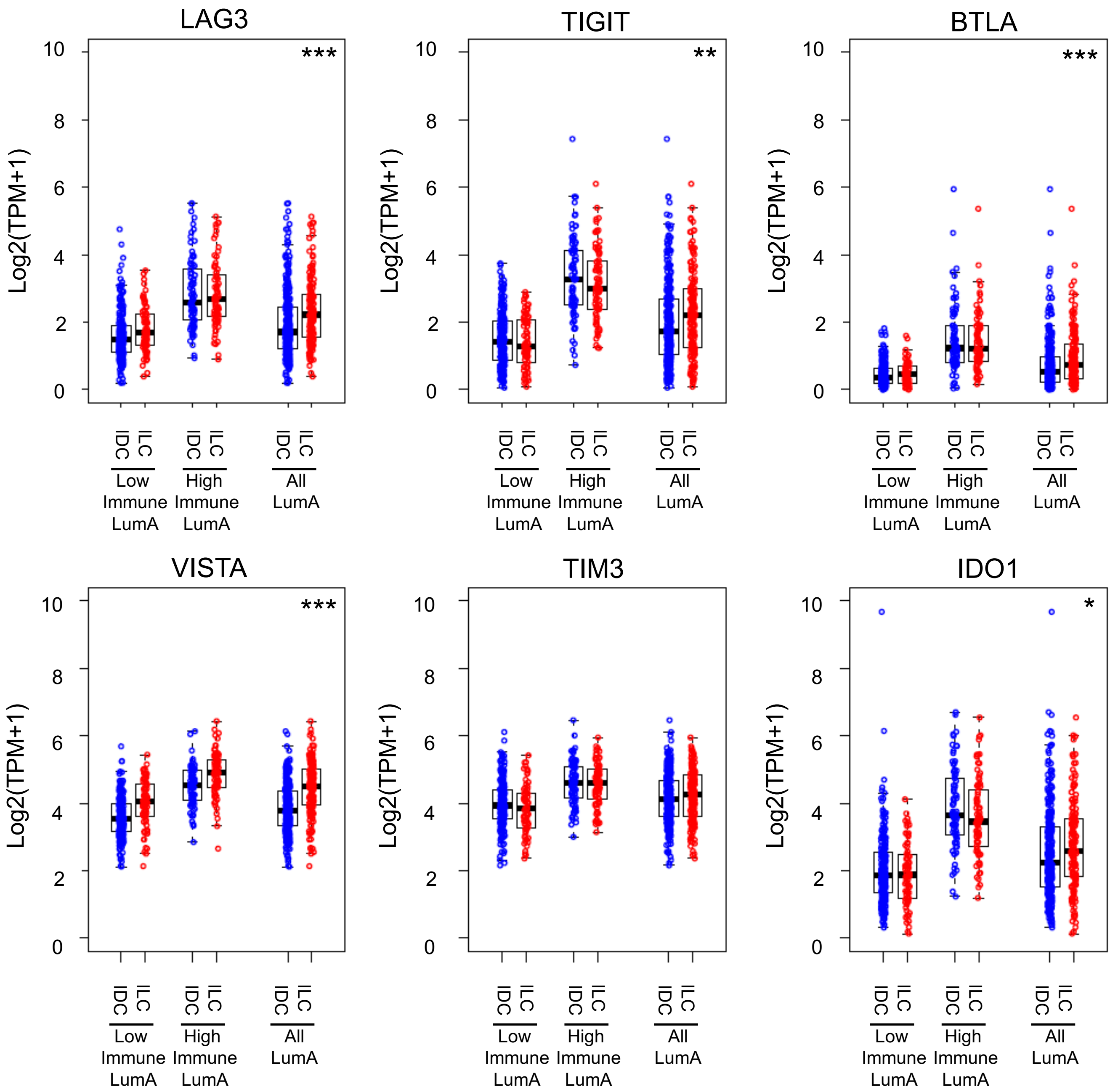

Figure S5: Differentially expressed genes and pathways with CPE correction.

a

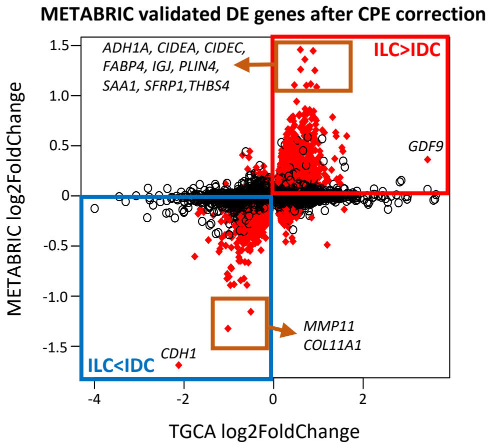

d

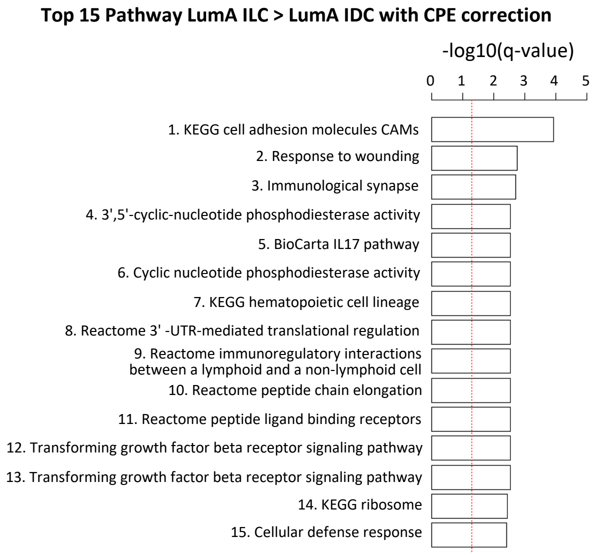

b

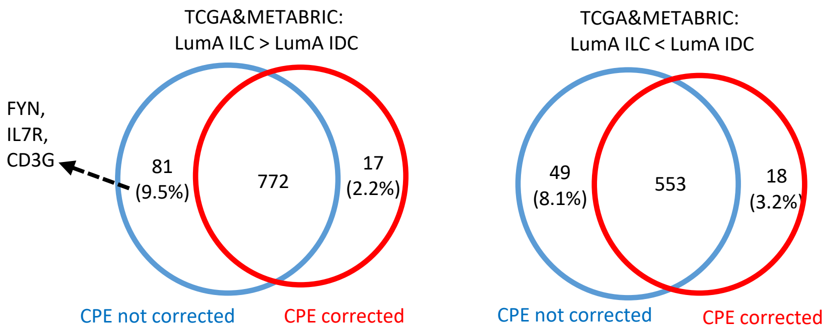

c

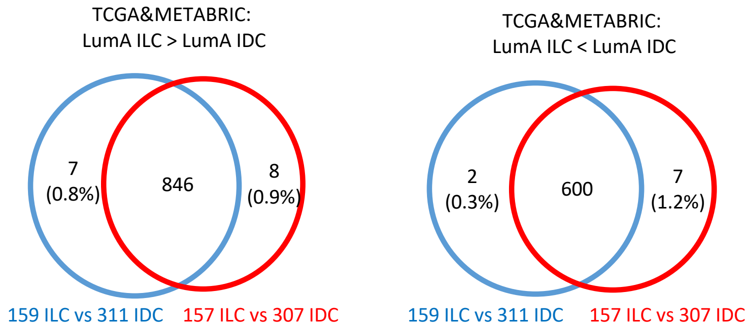

Figure S6: LumA ILC has lower protein/mRNA ratio than LumA IDC

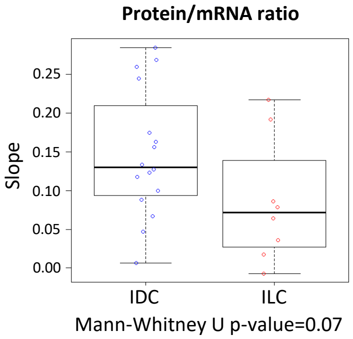

The following tables are available in additional excel files.

Supplementary table 2

TCGA tumor histology and PAM50 assignment

Supplementary table 3

METABRIC validated TCGA DE genes before and after CPE correction

Supplementary table 5

Pathway analysis before and after CPE correction (Fisher’s exact test and GSEA)

Supplementary table 6

Genes in immune cell signatures

Supplementary table 9

DE proteins in LumA ILC vs LumA IDC and ER+ ILC vs ER+ IDC (CPE corrected)

Supplementary table 10

The list of 156 proteins used in the calculation of protein/mRNA ratio

Supplementary table 1
Patient summary in TCGA, METABRIC, and Ciriello et al, Cell 2015 datasets.

	TCGA		METABRIC		Ciriello et al, Cell 2015	
ILC+IDC	N=971	%	N=1695	%	N=617	%
**Histology**						
ILC	197	20.29%	147	8.67%	127	20.58%
IDC	774	79.71%	1548	91.33%	490	79.42%
**ER**						
Positive	718	73.94%	1282	75.63%	440	71.31%
Negative	210	21.63%	392	23.12%	140	22.69%
**ILC PAM50**						
LumA	159	80.71%	65	44.22%	106	83.46%
LumB	18	9.14%	29	19.73%	6	4.72%
Normal	13	6.60%	38	25.85%	12	9.45%
Basal	2	1.02%	5	3.40%	1	0.79%
Her2	5	2.54%	9	6.12%	2	1.57%
**IDC PAM50**						
LumA	311	40.18%	533	34.43%	201	41.02%
LumB	202	26.10%	401	25.90%	122	24.90%
Normal	18	2.33%	120	7.75%	9	1.84%
Basal	162	20.93%	278	17.96%	107	21.84%
Her2	81	10.47%	212	13.70%	51	10.41%

Note: The percentage of each intrinsic subtypes in our assignment is similar to what's defined in Ciriello et al. [2] .

Supplementary table 4
Adjusted p-value of selected DE genes in survival analysis

	Log2FoldChange in TCGA	Log2FoldChange in METABRIC	LumA ILC P-value	LumA IDC P-value
ADH1A	0.7099	1.4590	0.9155	0.7336
CD36	0.3933	1.0495	0.8498	0.7336
CDH1	-2.1789	-1.6887	0.8498	0.7863
CIDEA	1.0873	1.0869	0.8498	0.9420
CIDEC	1.0578	1.4471	0.8498	0.7336
COL11A1	-0.9868	-1.3236	0.9155	0.5502
FABP4	0.7739	1.2617	0.9155	0.7336
GDF9	2.0855	0.3627	0.8498	0.7336
GRIA2	-0.7157	-1.0851	0.8498	0.7336
IGJ	0.6413	1.1060	0.8498	0.9420
KLK11	1.8255	1.1339	0.8498	0.5502
MMP11	-0.4760	-1.1558	0.9155	0.5502
PLIN4	0.9942	1.1162	0.9155	0.7336
SAA1	1.2003	1.3621	0.9155	0.7336
SFRP1	0.8354	1.2520	0.8498	0.2062
THBS4	0.8108	1.1017	0.9155	0.7336

Note: METABRIC survival analysis using median gene expression as cutoff. Log rank test p-values were adjusted by Benjamini-Hochberg method. All log2FoldChanges were not corrected with CPE.

Supplementary table 7
METABRIC validated TCGA DE genes in protein translation/regulation pathways.

Pathways	DE genes
Reactome 3' -UTR-mediated translational regulation	EIF3G<br>EIF3K<br>**RPL12**<br>**RPL13A**<br>**RPL18A**<br>**RPL31**<br>**RPL35A**<br>**RPL4**<br>**RPL5**<br>**RPL7A**<br>**RPS13**<br>**RPS27**<br>**RPS27A**<br>**RPS3**<br>**RPS3A**<br>**RPS6**
Reactome peptide Chain elongation	**RPL12**<br>**RPL13A**<br>**RPL18A**<br>**RPL31**<br>**RPL35A**<br>**RPL4**<br>**RPL5**<br>**RPL7A**<br>**RPS13**<br>**RPS27**<br>**RPS27A**<br>**RPS3**<br>**RPS3A**<br>**RPS6**

Note: Ribosome protein synthesis genes were marked in bold

Supplementary table 8
Alteration of proteins in PI3K/AKT/mTOR pathway (CPE corrected).

	LumA ILC vs LumA IDC		ER+ ILC vs ER+ IDC	
Protein	Log2FoldChange	Adjusted p-value	Log2FoldChange	Adjusted p-value
AKT	-0.077	0.18	-0.092	0.044
Phospho-AKT (Ser473)	0.094	0.38	0.2	0.014
Phospho-AKT (Thr308)	0.11	0.19	0.20	0.0053
AMPKα	0.0064	0.89	0.052	0.16
Phospho-AMPKα (The 172)	0.046	0.54	0.070	0.25
PI3K (p110α)	0.0038	0.90	0.0053	0.86
PI3K (p85)	0.054	0.17	0.079	0.015
mTOR	-0.063	0.12	-0.069	0.037
Phospho-mTOR (Ser 2448)	-0.089	0.00035	-0.082	0.00029

Note: LumA ILC (n=113), LumA IDC (n=242); ER+ ILC (n=135), ER+ IDC (n=411). P-values were adjusted by Benjamini-Hochberg method
